# Ultrasonic-assisted enzymatic extraction, physicochemical properties and prebiotic activities of polysaccharides from *Saccharomyces cerevisiae* spore wall

**DOI:** 10.1016/j.ultsonch.2025.107258

**Published:** 2025-02-08

**Authors:** Mengqing Yan, Guoyu Liu, Shiwei Liu, Jia Liu, Haizhi Li, Haotian Wang, Yan Zou, Cong Pan, Fang Zhou, Xueying Zeng, Youqiang Yu, Yimin Wu, Shuheng Yang, Shenglin Duan, Peng Yuan

**Affiliations:** a China National Research Institute of Food and Fermentation Industries, Beijing 100020, PR China; b College of Biological Science and Technology, Beijing Key Laboratory of Forest Food Processing and Safety, Beijing Forestry University, Beijing 100083, PR China; c College of Food Science and Engineering, Northwest A & F University, Yangling 712100, Shaanxi, PR China

**Keywords:** *Saccharomyces cerevisiae* spore, Polysaccharides, Physicochemical properties, Gut microbiota

## Abstract

Yeast wall polysaccharides (YWPs) are a bioactive compound found in the vegetative cells of *Saccharomyces cerevisiae*, contributing to its applications in food and medicine. YWPs have been extensively studied because of their diverse biological activities. Although the yeast spore wall of *S. cerevisiae* also contains polysaccharides, the biological activities of the components of the spores have received limited attention. In this study, yeast spore wall polysaccharides (YSWPs) were extracted from *chs*3Δ spores with exposed β-glucan layers via an ultrasonic-assisted enzymatic extraction (UAE) method, and the optimal extraction process was determined through single-factor and orthogonal experiments. The physicochemical properties of YWPs and YSWPs were compared under identical extraction conditions, and the effects of YSWPs on the abundance of the intestinal microflora and short-chain fatty acids (SCFAs) production *in vitro* were investigated. The results revealed that the optimal UAE process for YSWPs involves an ultrasonic power of 300 W, an ultrasonic duration of 60 min, an enzyme concentration of 1 % (w/w), and an enzymatic hydrolysis time of 4 h. The total sugar content of the extract was found to be 89.20 ± 0.52 %. The primary monosaccharides present in YSWPs are mannose and glucose, which are consistent with those found in the vegetative cell wall polysaccharides. Both YWPs and YSWPs exhibit similar physical properties, however, YSWPs has a smaller particle size, resulting in superior water-holding and oil-holding capacities. In simulations of *in vitro* colon fermentation, YSWPs can increased the population of beneficial microorganisms in the human intestine while reducing the number of harmful microbial populations. Additionally, it can effectively produced propionate and butyrate. These findings suggest that YSWPs can help maintain the intestinal ecological balance and promote intestinal health.

## Highlights

• YSWPs has a smaller particle size, leading to superior water holding capacity, oil holding capacity and swelling capacity.
• YSWPs can promote the growth of beneficial bacteria while inhibiting harmful microbiota.
• YSWPs can be metabolized by gut microbes, leading to the production of propionic acid and butyric acid.

## Graphical abstract

**Figure:**
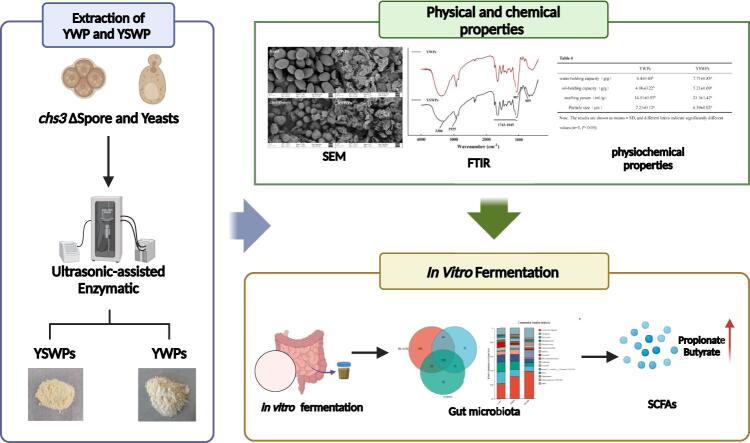

## 1 Introduction

*Saccharomyces cerevisiae*, known as baker's yeast, is recognized as a Generally Recognized as Safe (GRAS) microorganism [1]. It is rich in various nutrients, with cell wall polysaccharides being the primary active components. The cell wall polysaccharides derived from *S. cerevisiae* are mainly composed of α-1,6-mannan and β-1,3/1,6-glucan [2]. Specifically, α-mannan is present in the outer layer of the yeast cell wall, whereas β-glucan is located in the inner layer. Owing to the ease of scale-up in production, cell wall polysaccharides from *S. cerevisiae* have been systematically investigated, revealing a diverse array of physicochemical characteristics and pharmacological activities, including water holding capacity (which can affect food texture and stability), oil holding capacity (enhancing food emulsion stability), gut microbiota modulation, immunomodulatory effects, antidiabetic effects, and hypolipidemic properties [3], [4], [5], [6]. As a result, they have been extensively utilized in the food, pharmaceutical, and cosmetics industries [7], [8]. Notably, the spore wall of *S. cerevisiae* also contains β-glucan and α-mannan. However, few studies have focused on using spore wall polysaccharides, which warrants further investigation to explore their potential benefits and applications.

Yeast spores are haploid dormant cells formed by meiosis in *S. cerevisiae* under adverse conditions, such as nitrogen starvation and the presence of non-fermentable carbon sources (e.g., acetate) [9]. The primary components of the spore wall are also polysaccharides, but unlike vegetative cells, structural rearrangements during sporulation result in the β-glucan layer being located outside the mannan layer. The mature spore wall consists of four layers, from the innermost layer to the outermost layer: a mannan layer, a β-glucan layer, a chitosan layer, and a dityrosine layer [10]. Since the deposition of chitosan is an indispensable prerequisite for the formation of the dityrosine layer, mutant strains exhibiting deficiencies in chitosan synthesis, such as *chs*3Δ cells, are devoid of both the chitosan layer and the dityrosine layer. Notably, in *chs*3Δ spores, the β-glucan layer is the outermost layer of the spore wall. Recently, *chs*3Δ spores have been shown to possess anti-inflammatory and immunomodulatory properties, suggesting their potential applications in the food industry [11]. However, the physicochemical properties and physiological activities of polysaccharides in the *chs*3Δ yeast spore wall remain largely unknown.

Generally, the source of raw materials and the extraction method exert significant influence on the physicochemical properties, and bioactivities of natural polysaccharide [12], [13], [14], [15], [16]. Currently, yeast cell wall polysaccharides are typically extracted via autolysis or acid-base methods [17]. However, the use of acidic and alkaline can disrupt the hydrolyzable bonds of polysaccharides, Additionally, since spores have strong stress resistance and hight acid-base tolerance, make it challenging to extract spore wall polysaccharides via these methods. Ultrasonic-assisted enzymatic extraction (UAE) is a technique that uses high-frequency sound waves to generate cavitation bubbles within a liquid mixture of substrates and enzymes [18]. By combining the benefits of enzymolysis with the mechanical effects of ultrasound, this method has the potential to overcome some of the limitations associated with traditional cell disruption methods. Moreover, it effectively enhances the extraction efficiency of target polysaccharides while preserving their structural integrity and bioactivity. WANG et al. [19] found that the yield and anti-lipid peroxidation activity of polysaccharides extracted from lychee peel using UAE were superior to those extracted with an alkaline solution. Lin et al. [20] discovered that UAE extraction resulted in higher yield, sugar content and better antioxidant activity of Shatian pomelo peel polysaccharides (StPP) compared to traditional hot water extraction

Previously, various combined extraction methods have been carried out for cell wall polysaccharides extraction [21], [22]. However, effects of UAE on the extraction of *S. cerevisiae* spores wall polysaccharides have not been reported. Therefore, in this study, UAE was employed for the first time to extract polysaccharides from the cell wall of *S. cerevisiae* spores, and the optimal extraction process was established. Then the physicochemical properties of the spore wall polysaccharides (YSWPs) were assessed and compared with those of yeast cell wall polysaccharides (YWPs). Finally, the gut microbiota modulation effects during the *in vitro* fermentation of YSWPs were evaluated. The results provide a more sufficient scientific basis for the application of *S. cerevisiae* spores polysaccharides in food, medicine, and industry.

## 2 Materials and methods

### 2.1 Materials and reagents

The *S. cerevisiae* strains used in this study are SK-1 strain background. AN120 [23] was used as the wild-type strain. AN262 [24] was used as *chs*3Δ mutant cells, which was generously provided by the Hideki Nakanishi Research Group at the School of Biotechnology, Jiangnan University. Lyticase, Alkaline protease, Fuc, Rha, Ara, Gal, Glc, Xyl, Man, Gal-UA and Glc-UA were supported by Sigma-Aldrich (Shanghai) Trading Co., Ltd (Shanghai, China), Acetic acid, Propionic acid, Butyric acid standards were supported by Shanghai Aladdin Biochemical Technology Co., Ltd (Shanghai, China), Other chemicals are of analytically pure grades.

### 2.2 Preparation of spores

Yeast cells were cultured in YPAD (yeast extract 10 g/L, peptone 20 g/L, adenine 30 mg/L, glucose 20 g/L). YPAce (yeast extract 10 g/L, peptone 20 g/L, potassium acetate 20 g/L) and 2% potassium acetate medium were used for yeast sporulation.

Yeast cells derived from a single colony were cultured overnight in YPAD medium (yeast extract 10 g/L, peptone 20 g/L, adenine 30 mg/L, glucose 20 g/L) at 30℃. The cells were harvested by centrifugation, resuspended in 2% potassium acetate medium, and cultured for at least 24h. Cells were washed with water and suspended in water at 100 mg/mL. To release spores from asci, the ascal wall was digested with lyticase at the concentration of 625 U/g cells for 3 h at 37℃ with gentle shaking. Gradient centrifugation was performed to remove the lysed fragments, and the spores were finally collected by centrifugation after being washed with ultrapure wate.

### 2.3 Optimization of YSWPs extraction

#### 2.3.1 Single factor experiment

Take 0.5 g of spores or yeast cells into a centrifuge tube, add 5 mL of distilled water to make a suspension, then the suspension was treated with ultrasound at different power levels (60, 120, 180, 240, 300 W) for different times (20, 30, 40, 50, 60 min) by Ultrasonic Homogenizer (SCIENTZ, China). After sonication, centrifuge to collect the precipitate, wash with distilled water 3 times. Suspend the collected precipitate in distilled water, add different amounts of alkaline protease (0.5, 0.75, 1, 1.25, 1.5%, w/w) and enzymatically hydrolyze for different times (2, 3, 4, 5, 6 h). Collect the precipitate after enzymatic hydrolysis and lyophilize to obtain YSWPs and YWPs. The total sugar content [25] of precipitated and total protein content [26] of superserum were used as indexes for single factor analysis.

#### 2.3.2 Optimization of YSWPs extraction

An orthogonal L_9_(3)^4^ test design was used to investigate the optimal extraction condition of YSWPs. Based on a single factor test, ultrasonic power, ultrasonic time, enzyme concentration, and enzymatic hydrolysis time were arranged as three most influential moments.

### 2.4 Physicochemical Structure Characterization

#### 2.4.1 Particle size analysis

Particle size was conducted referred to the antecedent report [27]. 1 mg/mL YWPs or YSWPs solution was prepared and dispersed with ultrasonic treated. The particle size and particle size distribution were determined by wet method. The water refractive index was 1.33.

#### 2.4.2 Monosaccharide composition analysis

5 mg YSWPs sample was placed in ampoules with 3 mol/L TFA and hydrolyzed at 120℃ for 3 h. The acid hydrolyzed solution was accurately absorbed and transferred to the tube for nitrogen drying, then 5 mL water was added and mixed in a vortex, centrifuged at 12,000 rpm for 5 min, and the supernatant was diluted and then analyzed by ion chromatography IC. The chromatographic column was Dionex CarbopacTM PA20 (3×150 mm). Mobile phase: A: H_2_O; B:15 mmol/L NaOH; C: 15 mmol/L NaOH 100 mmol/L NaAc; flow rate: 0.3 mL/min; injection size: 25 µL; Column temperature: 30℃, electrochemical detector

#### 2.4.3 Fourier transform infrared spectroscopy (FTIR) analysis

2 mg samples and 200 mg potassium bromide were accurately weighed and pressed into tablets. The blank control was made by pressing tablets with potassium bromide powder. The samples were placed in FT-IR650 (Tianjin Gangdong China) for scanning and recording respectively

#### 2.4.4 Scanning electron Microscopy (SEM) analysis

The microstructure of cell wall polysaccharide samples was observed by scanning electron microscope Sigma 300 (ZEISS, Germany). YSWPs and YWPs samples were fixed to the sample rack with double-sided tape and imaged at an accelerated voltage of 3.0 kV with magnification of 5000 times.

### 2.5 Physiochemical properties

#### 2.5.1 Water holding capacity (WHC)

The WHC was determined according to the method of previous studies [28]. YWPs and YSWPs samples (0.2 g) were taken into a centrifuge tube, 10 mL distilled water was added, the test tube was mixed with a vortex every 5 min for 10 s for 30 min, and centrifuged at 1000 rpm/min for 15 min. The supernatant was discarded and the weight of the test tube and sediment was recorded, with the water capacity expressed as the weight of water adsorbed per gram of sample.

#### 2.5.2 Oil holding capacity (OHC)

Following the methodology of HUANG, et al [29] and modified appropriately, 0.2 g of YWPs or YSWPs samples was evenly mixed with 5 mL vegetable oil and placed at room temperature for 1h. The mixture was centrifuged at 8000 rpm for 15 min, and the sediment was weighed. The oil-holding capacity was expressed as the weight of oil adsorbed per gram of the sample.

#### 2.5.3 Swelling power (SP)

The SP was determined as described by JANTASON, et al [30] with slight modifications. The 0.2 g YWPs and YSWPs samples were hydrated with 10 mL distilled water in a calibrated cylinder at room temperature. After equilibrium for 12h, the bed volume was recorded. The swelling force was expressed as the volume occupied by the sample per grams of dry original sample (mL).

### 2.6 *In vitro* fecal fermentation

*In vitro* fecal fermentation was conducted based on the established methods [31]. The fecal samples were collected from healthy volunteers (3 women and 3 men) who did not have antibiotic treatment or digestive diseases for at least three months. *In vitro* fermentation was carried out in a 10 mL anaerobic fermentation flask. One milliliter of fecal slurry was mixed with 9 mL of the basal nutrient growth medium containing 100 mg of YSWPs or YWPs in an anaerobic chamber. The basal nutrient growth medium without any carbon source was used as the BLANK control. After fermentation for 0, 6, 12, 24, and 48h, the fermentation liquids were collected and stored at −80 °C until analysis.

#### 2.6.1 Microbiota composition analysis after *in vitro* fermentation

Total genomic DNA was extracted using a DNA extraction kit (DNeasy PowerSoil Kit, Qiagen). The V3-V4 variable region of 16S rRNA gene was amplified by PCR using upstream primer 338F (5′-ACTCCTACGGGAGGCAGCAGCAGG-3′) and downstream primer 806R (5′-GGACTACHVGGGTWTCTAAT-3′). Sequencing was performed at the Illumina HiSeq 2500 platform of the Shanghai Majorbio Bio-Pharm Technology Co., Ltd (Shanghai, China).

#### 2.6.2 Determining short-chain fatty acids (SCFAs) content

Referring to the method of previous studies [32], 800 μL SCFAs mixed standard solution or fermentation supernatant was thoroughly mingled with 200 μL 50% (w/v) concentrated sulfuric acid, then immediately added to 1 mL ether and vortexed for 1 min, centrifuged at 12,000 rpm/min for 10 min. The supernatant was filtered by 0.22 μm organic phase filter membrane and determined by gas chromatography (2010 plus Shimadzu Corporation, Japan). The carrier gas was N_2_ and the flow was 20 mL/min. Hydrogen flame ionization detector (FID) and the detector temperature was 250 ℃. The injection temperature was 240 ℃, injection volume was 1 μL. The heating procedure was to maintain80 ℃ for 0.5 min, raise the temperature to 150 ℃ at the rate of 4 ℃/min, followed by a ramp to 230 ℃ at 20 ℃/min, held for 10 min. The retention time and peak area of each peak were recorded, and the contents of SCFAs in each fermentation broth were calculated by standard curve.

### 2.7 Statistical analysis

All experiments were repeated three times in parallel. The results were expressed as mean ± standard deviation, statistical analysis was performed with SPSS 17.0, and GraphPad prism 9 was used for plotting analysis.

### 2.8 Process optimization study of YSWPs with USE method

**Fig. 1:**
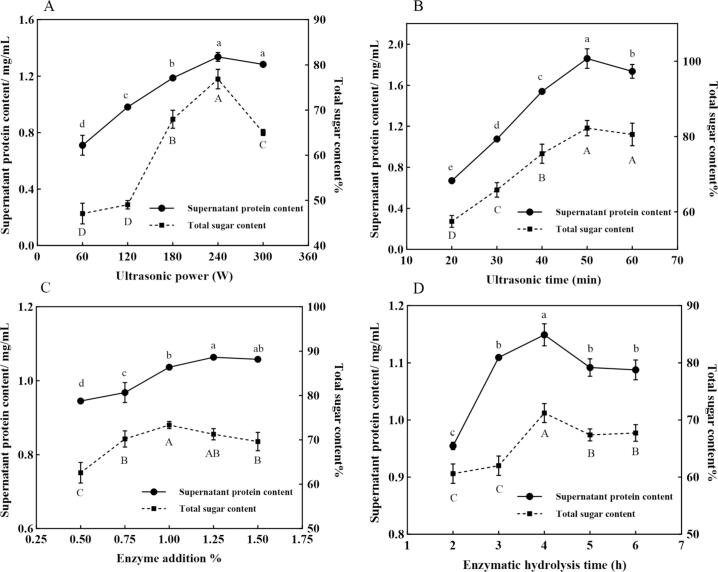
Effects of ultrasonic power (A), ultrasonic time (B), enzyme concentration (C), and enzymatic hydrolysis time (D).

As shown in Fig. 1A-B, at a constant ultrasonication time, the release of proteins and total sugar content increased progressively with higher ultrasonic power, indicating that ultrasonic power can facilitate cell lysis and increase extraction efficiency [33]. When the ultrasonic power was at 240 W, both the protein and total sugar contents decreased, suggesting complete cell disruption. This decrease may be attributed to a specific cavitation effect or a chemical reaction triggered by exceeding a certain threshold of ultrasonic power, which likely and breaks glycosidic bonds, leading to a reduction in polysaccharide content [20]. Under constant ultrasonic power and enzymatic hydrolysis conditions, extraction efficiency increased initially and then stabilized with longer ultrasonication durations. The optimal extraction efficiency was achieved at 50 min. Further ultrasonication disrupt ed the glycosidic bonds in polysaccharides, and fragmenting them into monosaccharides, thereby reducing the crude material polysaccharides content of the crude material [34].

As shown in Fig. 1C-D, during the progression of the enzymatic reaction, the cellular proteins gradually diffuse into the suspension. Under constant ultrasonication conditions and enzymatic duration, both the sugar content and soluble protein content in the supernatant increased with the addition of alkaline protease, with peak values observed at a final concentration of 1% (w/w). When the enzyme concentration was kept constant, peak levels were observed after four hours of enzymatic treatment, followed by a gradual decrease. This decline likely occurred because the enzyme fully reacts with the available substrate during the early stages of the reaction. Prolonging the reaction did not further increase the total sugar content [35]. Thus, the optimal central values for subsequent orthogonal experiment optimization are derived from the single-factor experiment analysis: ultrasound power, 240 W; ultrasonication time, 50 min; alkaline protease concentration, 1%; and enzymatic time, 4h.

Based on the results of the single-factor experiment, an L9(3^4^) orthogonal experiment was conducted to optimize the extraction process of cell wall polysaccharides. The factors and levels in the orthogonal design were shown in Table 1 and the experimental results were shown in Table 2, Table 3. From the analysis of the range, it can be concluded that the order of the factors affecting the total sugar content of the was ultrasonic power > enzymatic hydrolysis time > ultrasonic time > enzyme addition. The optimal process was A3B3C2D2: an ultrasonic power of 300 W, an ultrasonic time of 60 min, an enzyme concentration of 1% (w/w), and an enzymatic hydrolysis time of 4h. Variance analysis of the regression model revealed that, at a significance level of *P* < 0.05, the F-values for ultrasonic time, ultrasonic power, and enzymatic hydrolysis time were greater than the critical value of 19, indicating a significant effect on the experimental results. In contrast, enzyme concentration had a minor effect on the results. [36]. Under the optimal conditions obtained from the orthogonal experiment, the process was validated three times, and the validation experiment demonstrated that the total sugar content of the cell wall polysaccharide extract was 89.20±0.52%, which closely matched the result from the orthogonal experiment, confirming the reliability of the experimental results.

**Table 1:** Factors and levels in the orthogonal design.

Levels	Test factors			
	Ultrasonic power（W）	Ultrasonic time（min）	Enzyme concentration（%, w/w）	Enzyme hydrolysis time（h）
1	180	40	0.75	3
2	240	50	1	4
3	300	60	1.25	5

**Table 2:** The result of orthogonal experiment.

Test	Ultrasonic power A	Ultrasonic time B	Enzyme concentration C	Enzyme hydrolysis time D	Total sugar content%
1	1	1	1	1	60.07
2	1	2	2	2	68.07
3	1	3	3	3	70.61
4	2	1	2	3	81.66
5	2	2	3	1	74.65
6	2	3	1	2	90.85
7	3	1	3	2	85.68
8	3	2	1	3	82.50
9	3	3	2	1	84.26
K1	66.250	75.803	77.807	72.993	
K2	82.387	75.073	77.997	81.533	
K3	84.147	81.907	76.980	78.257	
R	17.897	6.834	1.017	8.540

**Table 3:** Analysis of variance table.

Factors	Sum of squares of deviation	Degree of freedom（df）	F-value	F-ratio	Significant
Ultrasonic power	583.780	2	333.018	19.000	*
Ultrasonic time	84.478	2	48.191	19.000	*
Enzyme concentration	1.753	2	1.000	19.000	
Enzyme hydrolysis time	111.371	2	63.532	19.000	*
Error	1.75	2			

Note: *P < 0.05.

### 2.9 Microscopic morphological observations of YWPs and YSWPs

**Fig. 2:**
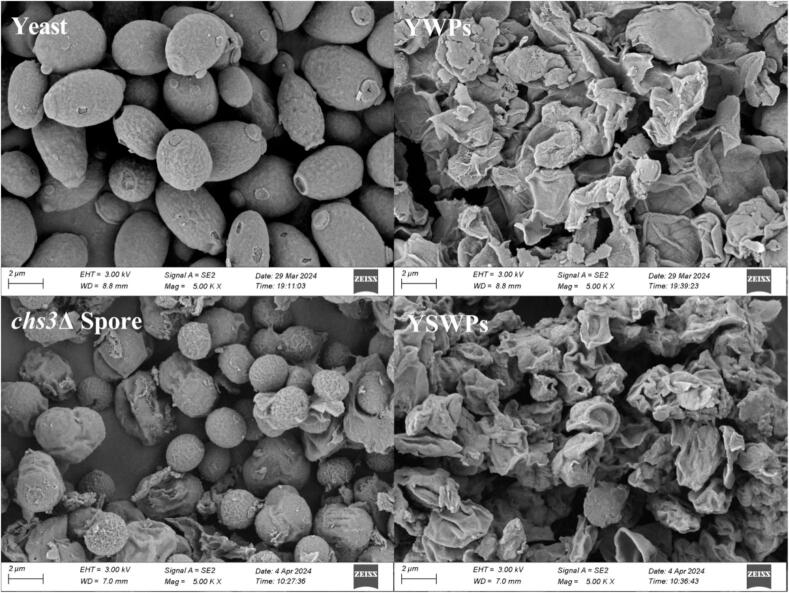
Scanning electronic micrographs of Yeast, YWPs, *chs*3Δ Spore and YSWPs (5000 × ).

Next, SEM was used to examine the microscopic morphological changes before and after extraction (Fig. 2). The original *S. cerevisiae* cells were elongate, ovoid, and had smooth surfaces, with a diameter of approximately 3–5 μm, In contrast, the *chs*3Δ spores were spherical, measuring around 2 μm, representing about one-quarter of the volume of the yeast cells. After UAE treatment, othe volumes of both yeast cells and spore cells were significantly reduced due to the solubilization of cytoplasmic content This reduction may be attributed to the loss of mannan glycoprotein [37].

### 2.10 Analysis of the monosaccharide compositions

**Fig. 3:**
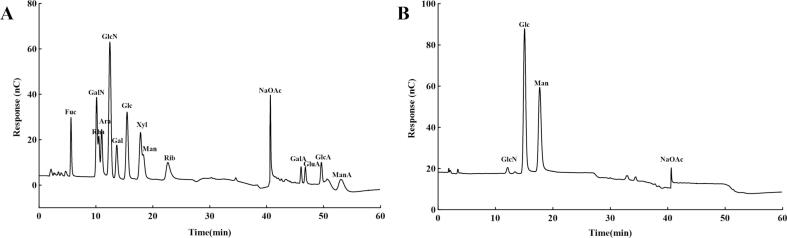
Monosaccharide compositions of YSWPs (A) Monosaccharide Standard, (B) YSWPs.

Fig. 3 shows that the primary components of YSWPs were mannose, glucose, and trace amounts of N-acetylglucosamine. The molar ratio of the three monosaccharides was 0.578: 0.402: 0.012, which is consistent with a previous report [38]. These findings suggest that YSWPs have a monosaccharide composition similar to that of yeast cell wall polysaccharides.

### 2.11 FTIR analysis

**Fig. 4:**
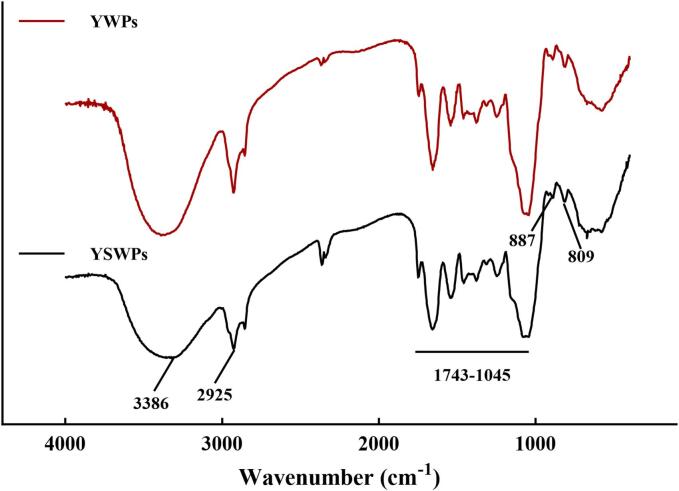
FTIR spectra of YWPs and YSWPs.

The FTIR analysis (Fig. 4) revealed that the infrared spectra of the two polysaccharides were similar. A broad and strong signal at 3386 cm^−1^ corresponds to the stretching vibration absorption of O-H, while the signals at 2925 cm^−1^, 2852 cm^−1^, 1743 cm^−1^, 1656 cm^−1^, and 1457 cm^−1^ represented the stretching vibrations of sugar C-H and C=O. The absorptions at 1076 cm^−1^ and 1045 cm^−1^ were characteristic absorption peaks of pyrans [6]. Furthermore, the angular vibration at 887 cm^–1^ corresponded to the characteristic absorption peak of the C-H deformation vibration of β-D-pyranose, indicating the presence of β-glucan in both samples. Additionally, the absorption peak at 809 cm^−1^ indicated the presence of α-mannose. This observation aligns with the previous structural studies on yeast cell wall polysaccharides by SHI et al. [39], suggesting that UAE is an effective method for extracting and preserving the structural integrity of spore cell wall polysaccharides.

### 2.12 Analysis of the physiochemical properties

WHC and OHC are important indicators for evaluating the functional properties of polysaccharides in the food industry. Polysaccharides with high WHC can retain more moisture in food, reducing shrinkage caused by dehydration and thereby enhancing product quality [40]. Additionally,polysaccharides with high OHC can enhance fat adsorption, reduce excess calorie intake, and help prevent obesity[41]. Table 4 presented the average particle size and the physicochemical properties of the YSWPs and YWPs. Under identical extraction conditions, YSWPs exhibited a smaller particle size ((4.39±0.02) μm) along with relatively higher WHC (7.71±0.83 g/g), OHC (5.21±0.69 g/g), and SP (21.30±1.47 g/g). Compared to YWPs, the WHC, OHC, and SP of YSWPs increased by approximately 20%, 28%, and 47%, respectively. Yeast cell wall polysaccharides are typically granular, with particle sizes similar to yeast cells, generally ranging from 3 to 10 μm [7]. However, since the volume of spores is significantly smaller than that of vegetative cells, the particle size of YSWPs is smaller than that of YWPs ((7.21±0.12) μm). Previous studies [42] have shown that the size of polysaccharides particles significantly influences their ability to bind water and oil. Smaller particle sizes result in larger specific surface areas, providing more binding sites for water molecules. Moreover it can increase porosity and capillary adhesion, thereby enhancing oil retention and binding capacity.

**Table 4:** Particle size, water-holding capacity, oil-holding capacity and swelling power of YWPs and YSWPs.

	YWPs	YSWPs
Water-holding capacity（g/g）	6.40 ± 0.44^b^	7.71 ± 0.83^a^
Oil-holding capacity（g/g）	4.06 ± 0.22^b^	5.21 ± 0.69^a^
Swelling power（mL/g）	14.51 ± 0.97^b^	21.3 ± 1.47^a^
Particle size（μm）	7.21 ± 0.12^a^	4.39 ± 0.02^b^

Note: The results are shown as means ± SD, and different letters indicate significantly different values (n = 3, *P <* 0.05).

LI et al. [43] found that the antioxidant capacity and immune function of yeast cell wall powder were closely associated with its particle size, with bioactivity significantly increasing as the particle size decreased. Compared to other methods, UAE treatment could reduces polysaccharide particle size and molecular weigh thereby enhance its *in vitro* biological activity[44]. Our study found that the spore wall polysaccharides obtained through UAE exhibited smaller particle sizes and higher water- and oil-holding capacities. Previous studies [45] have shown that the particle size of dietary fiber might play an important role in maintaining or recovering the balance and diversity of the microbiota community, suggesting that YSWPs extracted via UAE may have a promising potential to improve gut health. Therefore, in subsequent studies, we explored the prebiotic activities of YSWPs.

### 2.13 Effects of YWPs and YSWPs on the gut microbiota composition

**Fig. 5:**
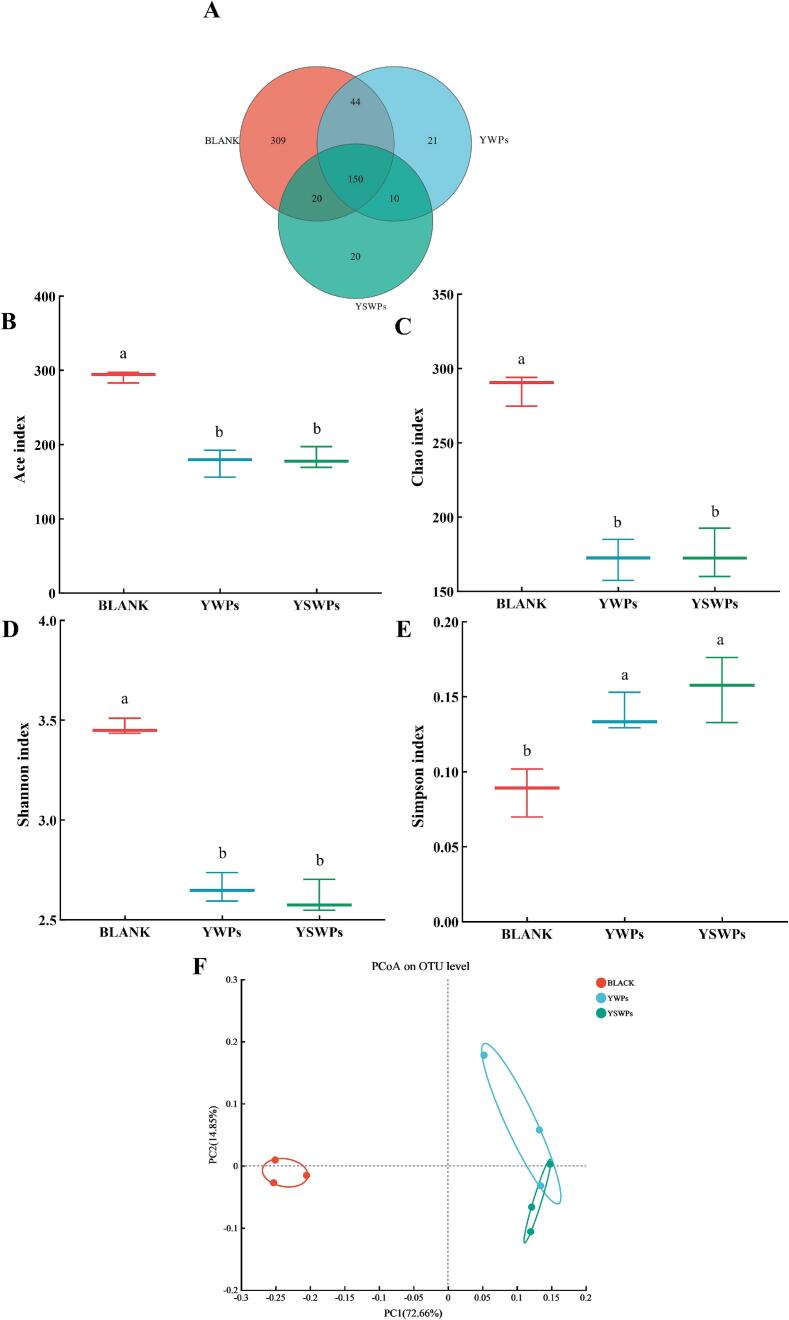
Venn diagram (A); Alpha diversity of samples among groups. B-E: Ace index (B), Chao index (C), Shannon index (D), Simpson index (E) and Principal coordinate analysis (PCoA) (F).

Human gut bacteria utilize polysaccharides to produce beneficial compounds, thereby promoting the body's energy metabolism and immune system stability. To investigate the effects of YWPs and YSWPs on gut microbiota diversity, high-throughput sequencing of 16S rRNA was performed, and the results are presented below. The generated sequences were clustered at the operational taxonomic unit (OTU) level with 97% similarity to reveal the composition of the gut microbiota. The Venn diagram illustrates the shared and OTUs among the different groups. As shown in Fig. 5A, the three samples were composed of total of 150 shared species, with YWPs and YSWPs containing 21 and 20 unique species, respectively, whereas the BLANK group included 309 unique species. These findings indicated that the inclusion of YSWPs altered the composition of the gut microbiome. One possible explanation for this phenomenon is that the yeast and its spore cell wall polysaccharides, or the substances produced during fermentation, may inhibit the growth of specific gut bacteria or may be inaccessible to certain microorganisms [31]. Alpha diversity was calculated to evaluate the richness and diversity of the microbial community and was characterized by four indices: the Ace index, Chao index, Shannon index, and Simpson index. As shown in Fig. 5B-E, the ACE, Chao, and Shannon indices for the YWPs and YSWPs groups were significantly lower than those of the BLANK group (*P* < 0.05), whereas the Simpson index was significantly higher in the YWPs and YSWPs groups compared to the BLANK group (*P <* 0.05)*.* These findings indicate that supplementation with YWPs or YSWPs may lead to an increase in the number of specific bacteria, thereby reducing the diversity and richness of the intestinal microbiota,. And the observations are consistent with the former observations of polysaccharides from *A. cristatus*[46] and polysaccharide from *Sparassis crispa* [47]. The beta diversity of the bacteria was evaluated through principal coordinate analysis (PCoA) score plot, as it shown in Fig. 5F. The YWPs and YSWPs groups were significantly different from the BLANK group, demonstrating that polysaccharide supplementation influenced the community composition of the intestinal bacteria. Overall, the results demonstrate significant differences in gut microbiota composition among the YSWPs, YWPs, and BLANK groups.

**Fig. 6:**
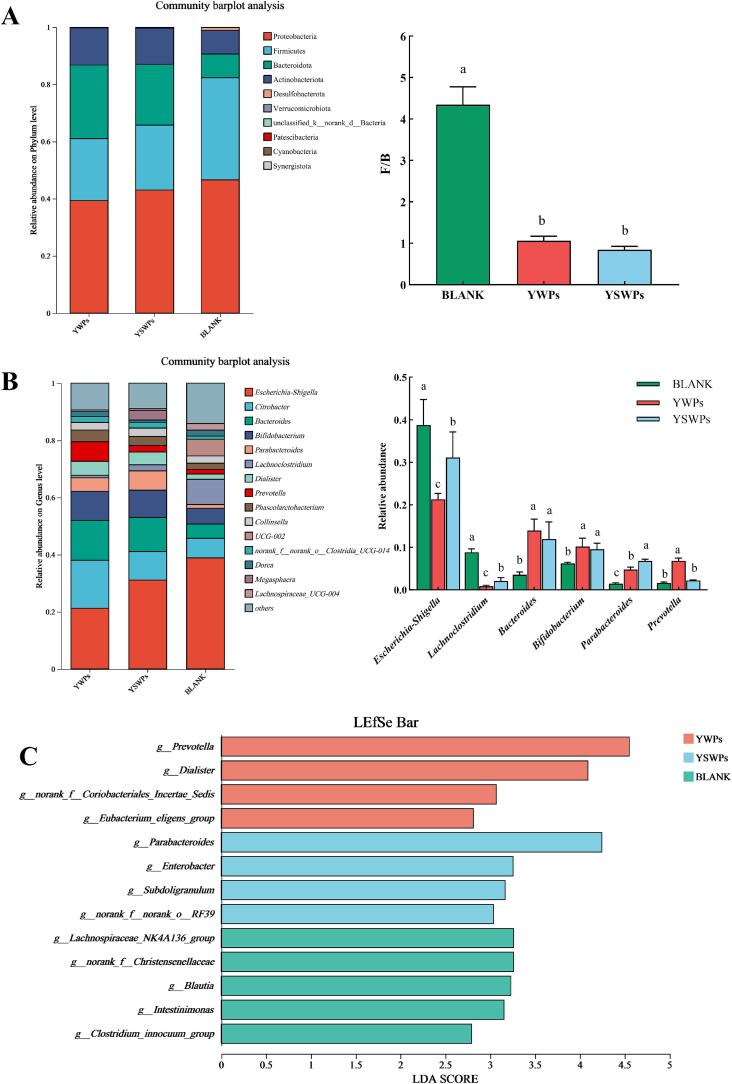
The relative abundance of bacterial community. A-B: at the phylum level (A) at the genus level (B) and comparison of microbiota based on linear discriminant analysis effect size LDA diagram (C).

The variations in the composition of the microbiota of the different groups during the *in vitro* fermentation at the phylum level were illustrated in Fig. 6A. Compared to the BLANK group, the groups incubated with YWPs and YSWPs showed a significant increased ratio of Bacteroidota and Actinobacteriota (*P <* 0.05). In contrast, the relative abundance of Firmicutes and Proteobacteria decreased (*P <* 0.05), which was consistent with the results of a previous study [48]. Proteobacteria are positively correlated with inflammation and diabetes, which are considered potential markers of dysbiosis and disease risk [49]. Bacteroides can produce SCFAs through the fermentation of indigestible carbohydrates, thereby providing health benefits to the host. These compounds serve as essential energy sources in the colon and play pivotal roles in immune regulation, antitumor activity, and antibacterial effects [50]. Furthermore, compared to the BLANK group, the proportion of Firmicutes to Bacteroidetes (F/B ratio) in the polysaccharide intervention group was lower. A reduction in the F/B ratio has been associated with decreased calorie intake, potentially supporting obesity risk management [51], [52]. Previous findings [52] have shown that obese individuals have a relatively high F/B ratio, which increases with the consumption of high-fat diets. These results suggest that YSWPs may have potential applications in obesity management and weight loss.

Fig. 6B presented a further analysis of the relative abundance of microorganisms at the genus level. Compared to the BLANK group, the abundances of *Escherichia-Shigella* and *Lachnoclostridium* were lower in the YWPs and YSWPs groups *(P <* 0.05*). Escherichia-Shigella* in the colon is associated with intestinal microbial dysbiosis, and its increased abundance can promote intestinal damage and disrupt amino acid metabolism, thereby contributing to intestinal microecological disorders [53]. *Lachnoclostridium* is an important bacterial genus in the colonic environment, and its relative abundance increases under conditions such as hepatic fatty degeneration and metabolic diseases, overabundance of *Lachnoclostridium* can lead to inflammation in the body [54]. Additionally, the abundances of *Bacteroides* and *Parabacteroides* increased in the polysaccharide intervention group (*P <* 0.05). Particularly, YSWPs exhibited stronger stimulation than YWPs on the proliferation of *Parabacteroide* (*P <* 0.05). Many studies have shown that these two genera can coordinate in the degradation of complex polysaccharides to gain a competitive advantage. This process assists the host in digesting long-chain polysaccharides and oligosaccharides that are not readily absorbed by colonic epithelial cells, thus enhancing intestinal digestive capacity and alleviating inflammation [55]. Furthermore, an increase in the abundance of *Bifidobacterium* was observed in both the YWPs and YSWPs groups (*P <* 0.05), *Bifidobacterium* are positively associated with intestinal health, which could degrade polysaccharides to promote the production of acetic acid, thus playing its part in preserving the intestinal barrier [56]*.* In summary, YSWPs can regulate gut health by inhibiting the proliferation of harmful bacteria (*Escherichia-Shigella*) and increasing the abundance of beneficial bacteria (*Bacteroides,* Bifidobacteria), making it a potentially effective functional dietary fiber.

To assess the differential microbiota among the fermentation groups, linear discriminant analysis effect size (LEfSe) was displayed applied to the OTU results. As shown in Fig. 6C, the YWPs group presented four genera-level differential species, whereas the YSWPs group presented six genera-level differential species. The genus with the greatest differential abundance in the YWPs group was *Prevotella*, whereas that in the YSWPs group was *Parabacteroides*. The yeast cell wall polysaccharides may vary depending on the yeast species or strain and composition of the culture medium [57]. Although *Saccharomyces cerevisiae* spores are dormant forms of yeast vegetative cells, their cell wall polysaccharides exhibit different particle sizes and functional characteristics due to structural recombination during their formation. This may be the important reason for the different regulatory effects of YSWPs and YWPs on the gut microbiota.

### 2.14 Changes in SCFAs

Functional oligosaccharides can reach the colon and be fermented by intestinal microorganisms, producing beneficial metabolic products such as SCFAs. These SCFAs can lower the pH of the colonic environment, altering intestinal acidity and helping to maintain physiological balance, thereby benefiting overall health [58]. As shown in Table 5, during fermentation, SCFAs levels in the YWPs and YSWPs groups were significantly greater (*P <* 0.05) than those in the BLANK group. After 24 h of fermentation, the total acid content in the YSWPs intervention group was significantly higher (*P <* 0.05) than that in the YWPs group. Additionally, propionic acid and butyric acid were identified as the main fermentation products. After 24 h of fermentation, the amounts of propionic acid in the YSWPs and YWPs group were 3.37-fold, and 2.63-fold of those in the BLANK group, respectively.And the butyric acid content were found for YSWPs of 1.73±0.02 μg/mL, YWPs of 1.51±0.06 μg/mL. Overall, YSWPs showed a higher increase in the content of butyrate and propionic acid than YWPs. Propionic acid can be transported to the liver via the portal vein, where it contributes to liver cell regeneration and inhibits cholesterol synthesis [59]. Butyric acid serves as a critical energy source for colonic epithelial cells, and has been demonstrated to play a role in colorectal cancer prevention and improving intestinal barrier function[60]. These observations reveal that YSWPs can be utilized by intestinal microorganisms in the colon to produce propionic and butyric acids, promoting intestinal health.

**Table 5:** Changes the content of SCFAs during *in vitro* fermentation.

SCFAs concentration (μg/mL)	Time (h)	BLANK	YSWPs	YWPs
Acetic acid	0	0.09 ± 0.01^c,A^	0.09 ± 0.01^d,A^	0.10 ± 0.01^d,A^
	6	0.23 ± 0.10^bc,A^	0.23 ± 0.04^c,A^	0.29 ± 0.01^c,A^
	12	0.38 ± 0.12^b,A^	0.5 ± 0.03^b,A^	0.48 ± 0.04^b,A^
	24	0.62 ± 0.01^a,B^	1.13 ± 0.01^a,A^	1.06 ± 0.10^a,A^
Propionic acid	0	ND	ND	ND
	6	0.31 ± 0.06^c,A^	0.33 ± 0.01^c,A^	0.4 ± 0.01^c,A^
	12	0.63 ± 0.11^b,B^	1.1 ± 0.12^b,A^	0.93 ± 0.03^bA^
	24	0.78 ± 0.17^a,C^	2.63 ± 0.30^a,A^	2.05 ± 0.01^a,B^
Butyric acid	0	0.07 ± 0.01^d,A^	0.05 ± 0.01^c,A^	0.07 ± 0.01^c,A^
	6	0.13 ± 0.07^bc,A^	0.13 ± 0.03^c,A^	0.19 ± 0.01^c,A^
	12	0.34 ± 0.32^b,A^	0.39 ± 0.15^b,A^	0.37 ± 0.01^b,A^
	24	0.92 ± 0.36^a,B^	1.73 ± 0.02^a,A^	1.51 ± 0.06^a,AB^
Total acid	0	0.13 ± 0.01^d,A^	0.1 ± 0.01^d,A^	0.17 ± 0.04^d,A^
	6	0.67 ± 0.09^c,B^	0.69 ± 0.02^c,A^	0.88 ± 0.01^c,A^
	12	1.35 ± 0.08^b,B^	1.99 ± 0.24^b,A^	1.78 ± 0.01^b,A^
	24	2.32 ± 0.18^a,C^	5.51 ± 0.33^a,A^	4.61 ± 0.03^a,B^

Note: a-d indicated that there were significant differences at different times in the same group *(P <* 0.05), and A-C indicated that there were significant differences among different groups at the same time *(P <* 0.05). ND- No detectable amount.

The production of SCFAs by microorganisms during carbohydrate fermentation is influenced by specific bacteria’s preference for and ability to utilize polysaccharides[47]. Propionic acid is primarily produced by species of the phylum Bacteroidetes, while butyric acid is mainly synthesized by a small number of Clostridia [61]. In the analysis of gut microbiota diversity, *Parabacteroides* was identified as the characteristic microorganism in the YSWP group, showing a higher degree of upregulation compared to the YWP group. This difference may explain the greater promotion of propionic acid production by YSWPs compared to YWPs.

## 3 Conclusions

In this study, the optimal extraction process for polysaccharides from spore walls was established through UAE. Our studies revealed that the polysaccharides extracted from the *chs*3Δ spore cell wall have similar physicochemical properties to those of yeast cell wall polysaccharides, but with smaller particle sizes and enhanced functional properties, making it more suitable for application in the food industry. During colonic fermentation, YSWPs can be utilized by colonic microbes, promoting the growth of beneficial bacteria such as *Bifidobacterium, Parabacteroides* and *Bacteroides*, while inhibiting the growth of harmful bacteria such as *Escherichia coli*, thereby improving gut health and maintaining the intestinal environment. In addition, YSWPs can produce propionate and butyrate, which are beneficial to gut microbiota, thus collectively promoting a healthy gut microenvironment. However, the conclusions drawn in this study are based on *in vitro* experiments and need to be further validated by in vivo studies. Future research will focus on using animal models to evaluate the effects of YSWPs on gut microbiota diversity, immune response, and intestinal barrier function. The result lays the foundation for the application of yeast spore wall polysaccharides as potential prebiotics in the field of gut health, while also providing new directions for the use of yeast spores and their components.

## CRediT authorship contribution statement

**Mengqing Yan:** Writing – original draft, Visualization, Investigation, Data curation, Conceptualization. **Guoyu Liu:** Writing – review & editing, Validation, Supervision, Conceptualization. **Shiwei Liu:** Supervision, Resources. **Jia Liu:** Resources, Project administration. **Haizhi Li:** Validation, Supervision. **Haotian Wang:** Validation, Methodology. **Yan Zou:** Investigation. **Cong Pan:** Supervision. **Fang Zhou:** Resources. **Xueying Zeng:** Resources. **Youqiang Yu:** Validation. **Yimin Wu:** Methodology. **Shuheng Yang:** Validation. **Shenglin Duan:** Writing – review & editing, Supervision, Funding acquisition, Conceptualization. **Peng Yuan:** Writing – review & editing, Supervision, Resources.

## Declaration of competing interest

The authors declare that they have no known competing financial interests or personal relationships that could have appeared to influence the work reported in this paper.
